# Demonstrative Midwifery, Again

**Published:** 1850-10

**Authors:** 


### DEMONSTRATIVE MIDWIFERY, AGAIN.

We stated in our last number, that the favorable opinion of this method of teaching, as once expressed by us, and since withdrawn, was based upon *erroneous information*. We wish it to be distinctly understood that that information was not derived from *any one connected with Buffalo Medical College, either directly or indirectly*.

